# Vaccine delivery using nanoparticles

**DOI:** 10.3389/fcimb.2013.00013

**Published:** 2013-03-25

**Authors:** Anthony E. Gregory, Richard Titball, Diane Williamson

**Affiliations:** ^1^College of Life and Environmental Sciences, University of ExeterExeter, UK; ^2^DSTL Porton Down, Salisbury, WiltsireSP4 0QJ, UK

**Keywords:** nanoparticle, vaccine, adjuvant, antigen-presenting cell, immunity

## Abstract

Vaccination has had a major impact on the control of infectious diseases. However, there are still many infectious diseases for which the development of an effective vaccine has been elusive. In many cases the failure to devise vaccines is a consequence of the inability of vaccine candidates to evoke appropriate immune responses. This is especially true where cellular immunity is required for protective immunity and this problem is compounded by the move toward devising sub-unit vaccines. Over the past decade nanoscale size (<1000 nm) materials such as virus-like particles, liposomes, ISCOMs, polymeric, and non-degradable nanospheres have received attention as potential delivery vehicles for vaccine antigens which can both stabilize vaccine antigens and act as adjuvants. Importantly, some of these nanoparticles (NPs) are able to enter antigen-presenting cells by different pathways, thereby modulating the immune response to the antigen. This may be critical for the induction of protective Th1-type immune responses to intracellular pathogens. Their properties also make them suitable for the delivery of antigens at mucosal surfaces and for intradermal administration. In this review we compare the utilities of different NP systems for the delivery of sub-unit vaccines and evaluate the potential of these delivery systems for the development of new vaccines against a range of pathogens.

## Nanoparticles as vaccine delivery vehicles

Traditional vaccines include live attenuated microbes, killed microbes, or components of microbes. Although many of these vaccines have been central to the control of infectious disease, some do not afford good protection against disease. In addition, some live vaccines are not safe for use in the growing population of immunocompromised individuals in society. There is also a wide range of infectious diseases for which no licensed vaccines are available. To address these challenges a range of vaccines are being developed based on isolated proteins or polysaccharides or naked DNA encoding a protective antigen. Whilst these can be safer, more defined, and less reactogenic than many existing vaccines, they are often poor immunogens, which require adjuvants to boost their efficacy. The most commonly used adjuvants are aluminium based but these can induce local reactions and may fail to generate strong cell-mediated immunity (Guy, 2007; Harandi et al., 2010). As a consequence, there is a great need to develop novel adjuvants and delivery systems for the next generation of vaccines.

Recently attention has been directed toward the utility of nanoparticles (NPs) as delivery vehicles for vaccines. The vaccine antigen is either encapsulated within or decorated onto the surface of the NP. By encapsulating antigenic material, NPs provide a method for delivering antigens which may otherwise degrade rapidly upon injection or induce a short-lived, localized immune response. Conjugation of antigens onto NPs can allow presentation of the immunogen to the immune systems in much the same way that it would be presented by the pathogen, thereby provoking a similar response. Moreover, NPs made from some composites enable not only site directed delivery of antigens but also the prolonged release of antigens to maximize exposure to the immune system. Also being explored is the potential for NPs to deliver vaccines through non-traditional methods such as topical, inhalation, or optical delivery as well as combining several antigens to the same particle so as to protect against more than one disease.

In this review we have considered VLPs, liposomes, ISCOMs, polymeric NPs, and non-degradable NPs as delivery systems for microbial proteins. The expectation is that the particulate vaccines generated using these technologies will be better at providing potent antigen-specific humoral and cellular immune responses and will allow next generation vaccines to be devised against a range of infectious diseases.

## Preparation of nanoparticles

**Figure 1:**
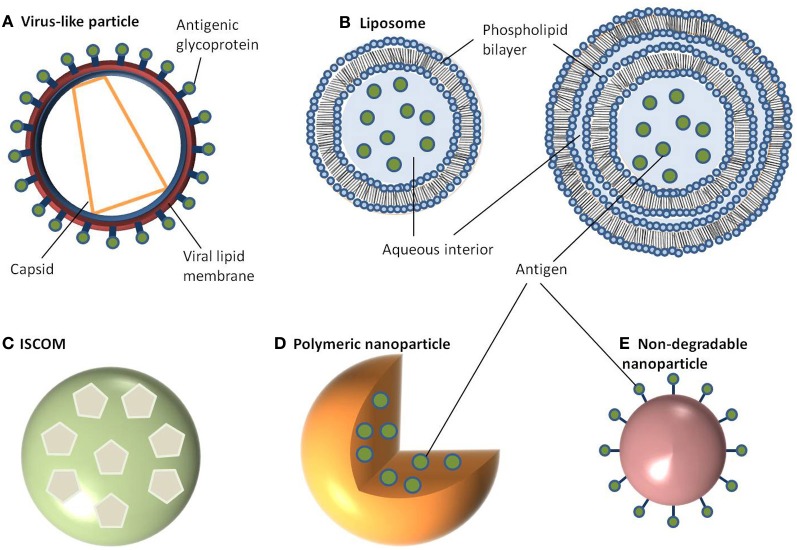
**Schematic representation of different nanoparticle delivery systems. (A)** Virus-like particle, **(B)** Liposome, **(C)** ISCOM, **(D)** Polymeric nanoparticle, **(E)** Non-degradable nanoparticle.

Amongst some of the first studied NP delivery systems are VLPs; attracting interest because of their ease of production and ability to stimulate strong immune responses (Kingsman and Kingsman, 1988; Roldao et al., 2010; Zeltins, 2012). Typically in the size range of 20–150 nm, VLPs consist of a self-assembled viral envelope, generated from a single protein to form a multimeric complex displaying a high density of epitopes (Grgacic and Anderson, 2006; Zeltins, 2012). Unlike viruses, VLPs assemble without encapsulating any viral RNA meaning they are non-replicating and non-infectious. Genes coding for viral integrase are also deleted prior to expression to prevent integration of the packed genome into the host cell and/or prevent recombination with live or defective virus in an infected individual (Young et al., 2006). VLPs can be engineered to express additional proteins either by fusing these proteins to the particle or by expressing multiple antigens (Kingsman and Kingsman, 1988; Strable and Finn, 2009). Using this approach, VLPs can be generated which provide protection not only against the virus of origin but also against heterologous antigens. Moreover, non-protein antigens such as polysaccharides or small organic molecules can be chemically coupled onto the viral surface to produce bioconjugate VLPs (Maurer et al., 2005; Patel and Swartz, 2011). The baculovirus expression system is most commonly used for the generation of VLPs, and has a good safety profile since baculoviruses do not naturally infect humans. The *Autographa california* multiple nuclear polyhedrosis virus (AcMNPV) is the most extensively studied VLP component (Hu, 2005). In this system a non-essential gene coding for the protein(s) forming the viral occlusion body (polyhedrin) is replaced with a gene of interest (Grgacic and Anderson, 2006). The vector encoding the modified VLP can then be used to infect insect cells (Sf9 or Sf21 derived from *Spodoptera frugiperda*, or BTI-TN-5B1-4 derived from *Trichoplusia ni*) to generate sufficient quantities of the viral protein which can then self-assemble into multimeric complexes (Figure 1A). The advantage of using such a system is that not only does AcMNPV have a large genome (130 kb), allowing for the insertion of multiple/large genes, but there is typically a high protein yield driven by the strong polyhedrin promoter (Hu, 2005). Despite its versatility, the main disadvantage to the baculovirus/insect expression system is its inability to produce authentic recombinant mammalian glycoproteins due to differences in post-translational modification patterns between insect and mammalian cell lines. One way to overcome this has been the development of “humanized” insect cell lines to constitutively express mammalian genes such as β1, 4-galactosyltransferase, and α2,6-sialyltransferase to enable the expression of terminally galactosylated and sialylated glycoproteins (Hollister et al., 1998; Jarvis et al., 2001; Aumiller et al., 2003; Jarvis, 2003; Harrison and Jarvis, 2006). Another problem associated with baculovirus expression system is the resulting cell death and lysis of insect cells within a few days after infection with baculovirus. This can be problematic for proteins which are selected for secretion or are vulnerable to degradation. Subsequent efforts to alleviate this problem have been in the form of a non-lytic baculovirus developed by random mutagenesis resulting in almost a 10-fold decrease in cell lysis and a reduction in degradation of expressed protein (Ho et al., 2004).

Like VLPs, liposomes are self-assembling but consist of a phospholipid bilayer shell with an aqueous core (Heurtault et al., 2010; Henriksen-Lacey et al., 2011). They can be generated as either unilameller vesicles, which consist of a single phospholipid bilayer, or multilameller vesicles, that are made of several concentric phospholipid shells separated by layers of water (Figure 1B). As a consequence, liposomes can be tailored to incorporate either hydrophilic molecules into the aqueous core or hydrophobic molecules within the phospholipid bilayers. There are a large number of methods published for preparing liposomes which are beyond the scope of this review (Riaz, 1996; Samad et al., 2007; Shailesh et al., 2009). However, typically these all involve a reverse phase evaporation process by dissolving phospholipids (such as monophosphoryl lipid A or phosphatidylcholine) in an organic solvent (e.g., chloroform, methanol). Water is then added, along with the antigen, and the solvent is evaporated resulting in large unilameller vesicles (Kersten and Crommelin, 1995; Zhu et al., 2005). Alternatively, liposomes can form in water by introducing a high energy input such as sonication, or nitrogen gas under high pressure. Initially this creates large multilameller vesicles, however, a continued energy input generates smaller, unilameller vesicles. Another method for preparing unilameller vesicles without subjecting antigens to high energy, which can sometimes be destructive, is by dissolving lipids in a detergent with a high critical micelle concentration, such as octylglucoside. The solution is then dialyzed against a buffer containing the antigen which results in the formation of liposomes (Kersten and Crommelin, 1995). In any of these methods, cholesterol can be (and often is) added to provide additional stability to the phospholipid bilayer. Other approaches to encapsulating antigens in liposomes include using repeat freeze thaw cycles (Zhu et al., 2005), a pH gradient (Waterhouse et al., 2005), or an ammonium sulphate method (Haran et al., 1993) with antigen encapsulation rates varying between 25 and 72% (Fries et al., 1992; Baca-Estrada et al., 2000; Zhao et al., 2007).

Colloidal saponin containing micelles of around 40 nm can be used as self-adjuvanting vaccine delivery systems and are collectively known as ISCOMs. Two types of ISCOMs have been described, both of which consist of cholesterol, phospholipid (typically either phosphatidylethanolamine or phosphatidylcholine) and saponin (most often Quil A from the tree *Quillaia saponaria*) (Kersten et al., 1988; Lovgren and Morein, 1988; Barr et al., 1998). Classically, ISCOMs have been used to entrap viral envelope proteins such as from herpes simplex virus type 1, hepatitis B, and influenza. However, proteins from a range of bacteria and parasites including *Escherichia coli*, *Brucella aborus*, and *Plasmodium falciparum* have also been used to assemble ISCOMs (Morein and Simons, 1985; Classen and Osterhaus, 1992; Morein et al., 1995). Complexes without viral proteins are also used and are often referred to as ISCOM matrices (Barr et al., 1998). ISCOMs are self-assembling at an optimal ratio of 1:1:5 (cholesterol:phospholipid:saponin) for matrices or 1:1:5:0.1/1 for classical ISCOM forming in the presence of a non-ionic detergent, which is then removed using dialysis or ultracentrifugation (Lovgren and Morein, 1988; Kersten et al., 1991). The resulting complex is a pentagonal dodecahedron arrangement of micelles containing saponin and lipid held together by hydrophobic interactions and stabilized through its negative surface charge (Figure 1C) (Özel et al., 1989; Kersten et al., 1991).

Polymeric NPs have attracted much attention for their ability to deliver drugs as well as being biodegradable (Li et al., 2000). Moreover, the release kinetics of loaded drugs from polymeric NPs can be controlled by compositional changes to the copolymer (Li et al., 2000). This class of NP can be prepared from a range of polymers including poly(α-hydroxy acids), poly(amino acids), or polysaccharides to create a vesicle which can either accommodate or display antigens. The most commonly used poly(α-hydroxy acids) for preparing polymeric NPs are either poly(lactic-*co*-glycolic acid) (PLGA) or poly(lactic acid) (PLA) which are often synthesized using a double emulsion-solvent evaporation technique (O'Donnell and McGinity, 1997; Sahoo et al., 2002; Lu et al., 2009). Firstly, a polymer of choice is dissolved in an organic solvent like ethyl acetate, ethyl acetate, or methylene chloride followed by the addition of the antigen which is then vortexed to get a primary emulsion. A water-in-oil-in-water emulsion is then formed with the addition of an emulsifying agent (e.g., polyvinyl alcohol or polyvinyl pyrrolidine). This results in the polymer precipitating around the antigen (Figure 1D). The solution is then left to allow solvent evaporation and then dried to prevent degradation of the polymer due to water-catalyzed ester hydrolysis (Sales-Junior et al., 2005; Feng et al., 2006; Pai Kasturi et al., 2006; Florindo et al., 2009; Harikrishnan et al., 2012). The use of this method is limited since antigen entrapment efficiency is low and there is a possibility of protein denaturation at the oil-water interface (Sah, 1999). The addition of stabilizers such as surfactants or sugars, including trehalose and sucrose, provide stability against denaturation by keeping the protein hydrated in its native form. An alternative method for retaining encapsulated protein stability uses poly(amino acids) such as poly(γ-glutamic acid) (γ-PGA), poly(ε-lysine), poly(L-arginine), or poly(L-histidine) which do not require an emulsion step in their synthesis (Lee et al., 2003; Matsusaki et al., 2004, 2005; Holowka et al., 2007). These amphiphilic copolymers self-assemble via hydrophobic interactions to form polymeric structures consisting of a hydrophobic core and a hydrophilic outer shell (Letchford and Burt, 2007; Lu et al., 2009). Moreover, γ-linked glutamic acids in γ-PGA are not easily recognized by common proteases resulting in added stability (Oppermann et al., 1998; Obst and Steinbuchel, 2004). To form these polymeric NPs, the poly(amino acid) is first dissolved in dimethyl sulfoxide (DMSO) before adding NaCl. The size of NPs is controlled by the concentration of NaCl resulting in monodisperse NPs ranging from 30 to 200 nm in diameter (Kim et al., 2009). To prepare protein-encapsulated γ-PGA NPs, the antigen (in saline) can be added to γ-PGA (in DMSO) and centrifuged (Akagi et al., 2011). The resulting encapsulation has between 30 and 60% efficiency and is stable over an acidic pH range even after 10 days (Akagi et al., 2011). Hydrophilic polysaccharide polymers are also good candidates for vaccine delivery with both dextran and chitosan being chosen for preparing NPs. Much attention has focused on chitosan NPs because of the biocompatibility of chitosan, its biodegradability into non-toxic products *in vivo* and its ability to open up tight junctions between epithelial cells (Sonaje et al., 2012). Chitosan NPs can be prepared in a number of ways. One method is a self-assembly technique through chemical modification, producing particles with a mean diameter of 160 nm (Lee et al., 1998). Similarly, a complex coacervation process is sometimes used whereby particles will spontaneously form when two hydrophilic colloids are mixed together, with chitosan precipitating around plasmid DNA (Mao et al., 2001). These particles are 100–250 nm in diameter and protect the DNA from nuclease degradation. The emulsion-droplet coalescence technique pioneered by Tokumitsu and colleagues was developed for intra-tumoral injection (Tokumitsu et al., 1999). It is based upon the emulsion crosslinking of chitosan and precipitation around the drug (gadopentetic acid). The particles formed were 450 nm in diameter and were appraised for their slow release and long-term retention within the tumor making them an excellent delivery vehicle. An ionic gelation process based on the positively charged amino groups in chitosan and the negative charge of tripolyphosphate has also been used to prepare chitosan NPs in the size range of 20–400 nm (Fernandez-Urrusuno et al., 1999; Xu and Du, 2003). Sometimes these colloids will be further modified by the addition of an adjuvant on the surface, such as polyethylene glycol in order to aid absorption or to slow down release.

**Figure 2:**
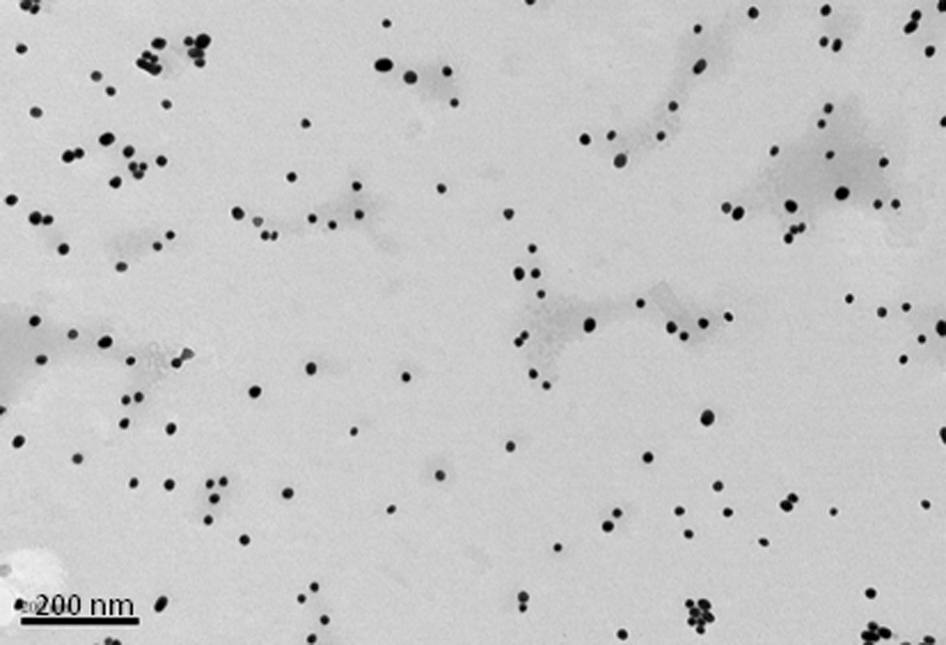
**Transmission electron micrograph of 15 nm gold nanoparticles formed when sodium citrate dihydrate is used to reduce gold(III) chloride trihydrate.** Bar is 200 nm.

In contrast to the above NPs, which consist of biological or biodegradable materials, non-degradable NPs are also being investigated for vaccine delivery (Calvo et al., 1997; Anne Saupe et al., 2006; Bhumkar et al., 2007b; Lee et al., 2010). Among those most commonly studied are gold, carbon, and silica to generate a shell in which to encapsulate antigens or, more commonly, to provide a surface for covalent attachment (Figure 1E). Gold NPs can vary considerably in size, but are frequently used in the 2–50 nm size range. Using chloroauric acid as the starting solution, the gold is reduced to form spherical particles of either 10–20 nm or 2 nm in diameter depending on whether a mild or strong reducing agent is used. In either case the particles formed are typically monodisperse and uniform in shape, which is essential for maintaining antigen loading consistency between batches (Figure 2). The smaller particles, formed from using a strong reducing agent, can then be grown to form larger particles with a desired aspect ratio using ceyltrimethlammonium bromide and silver acetate (Turkevich et al., 1951; Bhumkar et al., 2007a; Zhou et al., 2008; Chen et al., 2010). Carbon NPs have also been investigated for their use in vaccine delivery including oral delivery (Wang et al., 2011). Using silica NPs as a template, the particles are then carbonized at high temperatures under nitrogen gas and using sucrose as a carbon source. The resulting particle is over 450 nm in size with 50 nm mesopores embedded within the particle surface. Within these pockets a protein antigen can be protected from the harsh environment of the gastrointestinal tract, allowing oral administration to promote mucosal immunity (Wang et al., 2011).

Rather than delivery of whole microbes, as with traditional vaccines, the aforementioned NP delivery systems are focused on small molecular antigens produced by the pathogen. These components are usually expressed on the microbial membrane and may include polysaccharides, proteins, lipoproteins, glycoproteins (Table 1). In some cases DNA encoding microbial antigens has delivered and is then transcribed and translated in host cells, although these DNA vaccines are beyond the scope of this review. Membrane antigens used for vaccinations are often less immunogenic than whole microbes and require an adjuvant to boost the immune response. However, the safety profile of these small molecules is considerably better, making them much more attractive for future licensing of vaccines.

**Table 1:** **Summary of the various types of NPs currently being studied for their use as vaccine carriers**.

	**Matrix/expression system**	**Loading**	**Size**	**Antigen (pathogen)**	**Route of immunization**	**References**
Virus-like particles	Baculovirus (Sf9, Sf21, Hi5);	20–80μg	55–60 nm (HPV)	Major capsid protein, L1 (HPV);	Intramuscular, Subcutaneous,	Kang et al., 1999; Takamura et al., 2004; Villa et al., 2005; Giannini et al., 2006; Villa et al., 2006; Olsson et al., 2007; Quan et al., 2007; Sailaja et al., 2007; Slupetzky et al., 2007
	*E. coli*;	50μg	100–200 nm (HIV)	fms-like tyrosine kinase receptor ligand, FL (HIV);	Intraperitoneal,	
	Mammalian cells;	10μg	80–120 nm (H1N1)	gag precursor protein, pr45 (HIV);	Oral,	
	Yeast			HIV env cDNA (HIV);	Intranasal	
				Haemogglutinin (H1N1);		
				Nicotinamide (H1N1)		
				Matrix protein M1 (H1N1)		
Liposomes	MPLA;	1.26mg/ml	50–500nm	R32NS1 (malaria);	Intramuscular,	Alving et al., 1986; Fries et al., 1992; Baca-Estrada et al., 2000; Zhao et al., 2007; Karkada et al., 2011; Demento et al., 2012
(non-viral lipids)	Phospholipid S100 and cholesterol;	0.005mg/ml		Cholera toxin;	Intravenous,	
	Phosphatidylcholine and cholesterol	0.8–1mg/ml		Circumsporozoite (malaria);	Subcutaneous,	
		0.2 mg/ml		Lipid A;	Oral,	
				CtUBE fusion peptide (*H. pylori*);	Intranasal	
				KWC *Y. pestis*		
ISCOMs	Saponin (Quil A)	1–10μg	40 nm	HIV-1(gp120/160)	Intramuscular,	Kersten et al., 1988; Lovgren and Morein, 1988; Akerblom et al., 1993; Kazanji et al., 1994; Rimmelzwaan et al., 1994
	Phospholipid (phosphatidylethanolamine, phosphatidylcholine)	10μg		FIV(p130)	Subcutaneous,	
		30μg		*E. falciformis* (p27)	Oral	
	Cholesterol	100–500μg				
	Viral proteins					
Non-degradable	Gold;	1mg/ml	2–150nm	Plasmid DNA expressing haemagglutinin 1 (Influenza);	Intradermal,	Fynan et al., 1993; Bhumkar et al., 2007a,b; Bharali et al., 2008; Caputoa et al., 2009; Chen et al., 2010; Wang et al., 2011
	Silica;		5–470nm	Hepatitis B	Intramuscular,	
	Carbon				Subcutaneous,	
					Intravenous	
Polymeric	Poly(lactic-*co*-glycolic acid) (PLGA);	42.5mg/ml	100–200nm	Docetaxel;	Intramuscular,	Musumeci et al., 2006; Dhar et al., 2009; Demento et al., 2012
		10–50μg/ml	800nm	TetHc (Tetanus);	Intravenous	
	Poly(lactic acid) (PLA);		1–5μm	Hepatitis B;		
	Poly(glycolic acid) (PGA);		248 nm	SBm7462 (*Boophilus microplus*);		
	Poly(hydroxybutyrate) (PHB);			Rv1733c (*M. tuberculosis*);		
	Chitosan;			SPf66 (*P. falciparum* malaria)		
				Dtxd (Diptheria)

## Characterization of nanovaccines

Once synthesized, it is essential to characterize the structure and composition of NP formulations to avoid any variation between (or within) batches. Variation could arise from contamination, a polydisperse population of NPs, the accumulation of toxic components or incomplete particle formation. In order to maintain a homogenous population, several methods are employed to measure uniformity within colloidal solutions. Spatial uniformity amongst NPs is essential since the spherical volume will influence how much antigen is encapsulated or conjugated onto the surface and could vary the immunizing dose of the vaccine. Consequently, the size and shape of particles is characterized using a variety of methods including electron microscopy, dynamic light scattering, and density gradient centrifugation (Morein et al., 1984; Kersten et al., 1988). The amount of antigen-present is then quantified using one or more of the following techniques: Lowry and Bradford assays, enzyme-linked immunosorbent assay, dot-blots, density gradient centrifugation, sodium dodecyl sulphate polyacrylamide gel electrophoresis, and Western blotting (Carol et al., 1989, 1997; Erturk et al., 1991; Browning et al., 1992; Reid, 1992). In some instances it may be necessary to measure the compositional content of the NP if some of the reagents are toxic in high doses. This is especially true of Quil A, a key component of ISCOMs, which can have haemolytic effect in sufficient concentrations, and is measured in a rocket electrophoresis assay or by reversed phase high-performance liquid chromatography (Kersten et al., 1988; Sundquist et al., 1988). Other component of ISCOMs, such as cholesterol and phospholipids, are measured by gas chromatography and phosphorus assays respectively (Kersten et al., 1988). Quantification of metal (and non-metal) NPs, such as gold, can be quantified using instrumental neutron activation analysis or inductively coupled plasma mass spectrometry (Hillyer and Albrecht, 2001; Harkness et al., 2010).

## Vaccine induced immunity

For more than 70 years adjuvants have been added to vaccine formulations to boost the immune response to the vaccine. In total there have been several hundred natural and synthetic compounds identified as adjuvants, the most commonly used include an oil in water emulsion with and without the addition of mycobacteria (Freund's complete and incomplete adjuvant, respectively), lipid A from the lipopolysaccharide of Gram-negative bacteria and unmethylated cytosine-guanine dinucleotides (CpG) found in bacterial DNA. Despite this, aluminium-based compounds (principally aluminium phosphate or hydroxide) remain the most widely used adjuvants incorporated into licensed human vaccines. Until recently the mechanisms by which alum potentiates the immune response has been poorly understood and was initially believed to be due to a depot effect at the site of injection prolonging exposure of the antigen to the immune system for a better response (Glenny et al., 1931; Harrison, 1935). This theory was later challenged by Holt who showed that excision of the injection site from guinea pigs did not interfere with the development of a humoral response (Holt, 1950). Recent studies have also documented the rapid release of antigens from alum adjuvants; ~80% of aluminium phosphate adsorbed tetanus toxoid had disappeared from the site of injection within 4 h (Weissburg et al., 1995; Gupta et al., 1996). It is now believed that alum plays a more active role from observations of its electrostatic interaction with lipopolysaccharide (Shi et al., 2001); or its demanding effect on some protein antigens (Soliakov et al., 2012) leading to corrugated layers of aluminium oxyhydroxide held together with hydrogen bonds. Aluminium gel particles are generally no more than 10 μm in diameter, and antigens adsorbed to these particles maybe phagocytosed more readily than those without alum (Powell et al., 1995). The generation of particulate molecules *in vivo* may create an inflammasome which in turn may activate the Nlrp3 (NOD-like receptor family, pyrin domain containing 3) (Li et al., 2008; Eisenbarth et al., 2009) causing an influx of eosinophils and an upregulation of MHCII expression and antigen-presentation cell activity (McKee et al., 2009). Once activated, this cytoplasmic Nlrp3 protein promotes the production of pro-inflammatory cytokines such as interleukin 1beta and interleukin 18 (IL-1β and IL-18) (Li et al., 2007; Eisenbarth et al., 2009). Of these cytokines IL-1β has been shown to be a potent stimulus for T-cell dependent antibody production *in vivo* (Nakae et al., 2001). Alternatively, prostaglandins or other moieties may mediate the inflammatory response to the alum (Pelka and Latz, 2011). Although alum is renowned for its ability to produce antibody responses, it induces strong CD4-mediated cellular responses (predominantly Th2 but also Th1) and can also induce CD8 activation (McKee et al., 2009). Stimulation of these cellular responses induces cellular memory in man which is important for protection against many pathogens as well as generating long-term protective immunity (Bomford et al., 1992; Comoy et al., 1997; Hogenesch, 2002). Whilst alum-based adjuvants are generally well tolerated, there may be some associated toxicity problems such as the formation of granulomas when subcutaneous or indradermal injection is preferred over intramuscular; allergenicity and accumulation of aluminium if renal function is poor which not only can become highly toxic but has also been associated with amyotrophic lateral sclerosis and Alzheimer's disease (Straw et al., 1985; Goto et al., 1993; Gupta et al., 1996; Campbell, 2002). Consequently, there is an urgent need to develop safe adjuvants which are able to stimulate both Th1 and Th2 immune responses.

**Figure 3:**
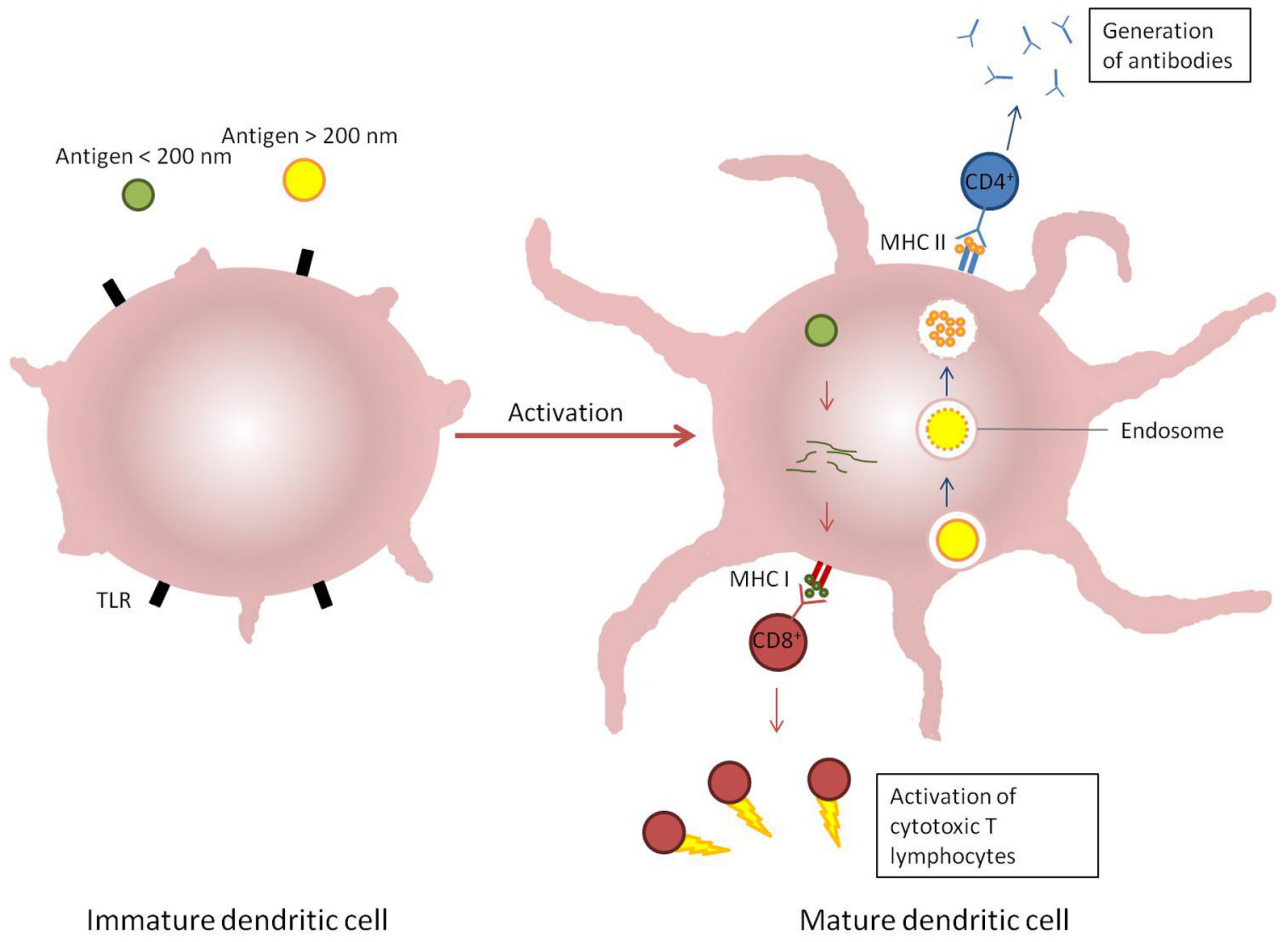
**Induction of immune response by dendritic cells response to different stimuli.** Antigens which enter cells via endosomal pathways (blue arrows) are typically degraded within a vesicle before the contents is displayed on the cellular surface by MHC II receptors and recognized by CD4^+^ T cells. Alternatively, antigens present in the cytosol (red arrows) are broken down and presented on MHC I receptors, which are recognized by CD8^+^ T cells.

The objective of vaccination with any formulation is to emulate the innate and adaptive responses of the immune system to infection (Bachmann and Jennings, 2010). The predominant interface between the innate and adaptive immune responses is antigen-presenting cells, and particularly dendritic cells (O'Hagan, 1998; Storni et al., 2005). Antigen-presenting cells are able to recognize micro-organisms through pattern recognition receptors such as toll-like receptors (TLR). On recognition of microbial surface determinants, antigen-presenting cells undergo maturation leading to a redistribution of MHC molecules from intracellular compartments to the cell surface, secretion of cytokines and chemokines, cytoskeleton reorganization, and morphological changes including the proliferation of dendrites from the membrane of dendritic cells. The micro-organism can be engulfed by the antigen-presenting cells through an endocytic pathway (phagocytosis) where it is typically degraded by proteolytic enzymes and reactive oxygen species. The peptides released by processing of proteins are then displayed on MHC class II molecules and are recognized by CD4^+^ T cells to stimulate the production of antigen-specific antibodies and the formation of memory T-cells. CD4^+^ T-cells are further divided functionally on maturation into Th1 or Th2 cells; induction of the former leads to a predominantly pro-inflammatory response with the secretion of interferons (typically IFNγ) and tumor necrosis factor α (TNFα), whereas the predominantly anti-inflammatory role of Th2 cells is to secrete cytokines such as IL4, IL10, and IL13. Both types of Th cell also support the production of antibodies by B-cells, in either a pro-or anti-inflammatory environment, which in turn influences antibody isotype and function. However, some pathogens such as viruses and some bacteria are able to become internalized within cells via non-endocytic pathways. When this occurs, antigens derived from the pathogen are processed via proteosomes which then display peptides in the clefts of MHC class I molecules (Shen et al., 2006). The displayed antigen is recognized by CD8^+^ T cells which have cytotoxic activity toward other host cells infected by the pathogen (Figure 3). In practice, the response to any pathogen may encompass a mix of all these, further complicated by the induction of pro-inflammatory Th17 cells and constrained by regulatory T-cells, but a predominant polarity (Th1 or Th2) may be required to resolve the infection (Bot et al., 2004).

## Nanoparticle uptake and immunity

It is important to be able to tailor vaccine-induced immunity to an appropriate response to deal with the pathogen. Moreover, the delivery of antigens to dendritic cells is central to the development of a protective immune response. Using NPs to deliver antigens, the efficiency of uptake into dendritic cells is significantly increased compared with soluble antigen alone; in some instances a 30-fold increase in uptake can be achieved (Akagi et al., 2011; Uto et al., 2011). Similarly, studies comparing differences in uptake between micro- and nano-PLA particles have found that uptake by antigen-presenting cells is significantly increased for NPs. Chithrani et al. investigated the dependency of gold NPs size on uptake into HeLa cells by incubating cells with a range of NP sizes (14–100 nm) and then measuring their gold content using inductively coupled plasma atomic emission spectroscopy. The results showed that the optimal size for uptake was 50 nm and uptake increased significantly for the first 2 h before plateauing at between 4 and 7 h post-exposure (Chithrani et al., 2006). Particle shape and surface charge are also important physicochemical factors playing crucial roles in the interaction between particles and antigen-presenting cells. In general, cationic particles are taken up into cells much more readily than those with an overall negative surface charge due to the anionic nature of cell membranes, whilst spherical as opposed to rod-shaped particles are also more readily endocytosed (Merdan et al., 2002; Foged et al., 2005; Xia et al., 2009).

As well as the degree of uptake, the mechanisms by which NPs enter cells will have a direct impact on the type of immune response induced. This too is dependent on NP size, as well as their composition, shape and charge, resulting in antigens being taken up into different intracellular trafficking pathways. Whilst PLGA microparticles typically enter macrophages through phagocytosis, there are a variety of mechanisms by which NPs may be internalized. It has been suggested that 43 nm polymeric NPs are taken up by HeLa cells via clathrin-dependent endocytosis, whilst 24 nm particles enter a cholesterol-independent, non-clathrin, and non-caveolar dependent pathway (Lai et al., 2007). NP shape can have a significant effect on the ability of macrophages to internalize particles via actin-driven movement of the macrophage membrane. Subsequently the phagocytosis of rod-shaped particles is often negligible when compared to spherical NPs (Champion and Mitragotri, 2006, 2009). Both polymer and gold cationic NPs have been shown to enter various mammalian cell lines via non-endosomal pathways using a range of pharmacological inhibitors or cell lines with endogenous proteins considered essential for a transport mechanism knocked-out (Ivanov, 2008; Sharma et al., 2010; Taylor et al., 2010; Vercauteren et al., 2010; Dos Santos et al., 2011; Iversen et al., 2011).

When poly(amino acid) NPs with encapsulated ovalbumin were used to immunize mice, significantly higher levels of total IgG, IgG1, and IgG2a were induced compared with the response to soluble ovalbumin, suggesting the particles have the ability to prime humoral and cellular immune responses since CD4^+^ and CD8^+^ T cell activation produces IFNγ which induce Ig class switching to IgG2a (Uto et al., 2007, 2009; Mohr et al., 2010). Similarly, the loading of Hepatitis B core antigen into PLGA NPs (300 nm) induced a stronger cellular immune response in a murine model than when Hepatitis B core antigen was administered alone. Particle size also plays an important role in directing the immune response. Immunization with PLA NPs (200–600 nm) was associated with higher levels of IFNγ production related to a Th1 response. In contrast, immunization with PLA microparticles (2–8 μm) promoted IL-4 secretion related to a Th2 response (Gutierro et al., 2002). Both PLGA NPs and liposomes are efficiently phagocytosed by dendritic cells in culture, resulting in their intracellular localization (Lutsiak et al., 2002; Copland et al., 2003; Elamanchili et al., 2004).

VLP's have been shown to produce strong humoral immune responses that are able to protect against human papillomavirus (HPV) infection in both animal models and human clinical trials using the HPV L1 protein (Breitburd et al., 1995; Kirnbauer et al., 1996; Koutsky et al., 2002; Harper et al., 2004; Villa et al., 2005). Through mimicking the native viral structure, VLP-based vaccines (including those against influenza A and HIV) are able to enhance the production of neutralizing antibodies by presenting antigens in their natural state as membrane-bound proteins rather than soluble ectodomains (Kemp et al., 2011; Pushko et al., 2011). However, this is mostly type-specific and may not protect against infection with heterologous types. Furthermore, cell-mediated immune responses were also achieved with HPV VLPs, including T cell proliferation (CD4^+^ and CD8^+^) (Emeny et al., 2002; Pinto et al., 2003). There is also an association of increases in Th1 and Th2 type cytokines (IFNγ and IL-5, IL-10 respectively) stimulated with VLP immunization (Evans et al., 2001; Pinto et al., 2003). Once *in vivo*, particulate vaccine formulations of all types constitute an antigen depot, the effect of which is to allow a gradual release of antigen, prolonging exposure of the immune system to the antigen and essentially providing a booster dose. The pharmacokinetics/pharmacodynanics of each formulation will determine how slowly or otherwise antigen is released from the depot. In general though, particulate formulations confer benefits in terms of a reduced need for the administration of booster doses and a self-adjuvanting effect due to the enhanced uptake of particulates by antigen-presenting cells.

## Limitations and ongoing questions

The limitations of NPs for the delivery of vaccines range from concerns over the toxicity of the particles, to difficulties in producing the materials and presenting antigens in their native form.

The production of suitable NPs can present some technical challenges. For example, although insect cell lines are widely used to express VLPs, they are unable to glycosylate proteins in the same way as mammalian cells. Consequently, some insect cell lines have been mammalianized to accommodate this (Palmberger et al., 2012). For other systems there are concerns over the stability or potential to scale-up production. One of the greatest obstacles with liposome delivery systems is their instability (Soppimath et al., 2001; Hans and Lowman, 2002). One of the ways in which this has been overcome is by modifying the surface with a hydrophilic polymer, such as glycol (e.g., polyethylene glycol, glycol chitosan). This serves as a barrier to reticuloendothelial system cells to extend its circulatory lifetime (Goren et al., 2000; Filipovic-Grcic et al., 2001). The scale-up of production of sterile polymeric particles has also been problematic, though this has to some extent been overcome by the introduction of scaled-up spray-drying techniques. This allows polymer and payload, together with a stabilizer such as trehalose, to be sprayed at high temperature (80–100°C) through an orifice in clean room conditions (Baras et al., 2000; Varshosaz et al., 2011). This is a batch process, typically yielding hundreds of milligrams of product (depending on equipment size and operating conditions), but care needs to be taken that a protein payload will not be damaged by either the heat or shear force. One way around this is to spray -dry the polymer and subsequently surface-absorb the protein antigen(s). Additionally, in order to overcome the risk of traces of unacceptable solvents in the final NP product, super-critical fluid technology is being applied to the generation of NP (Valle and Galan, 2005; Vemavarapu et al., 2005). In this approach, the PLGA polymer is solubilized in super-critical fluid solvent (freon/propanol) and the solution is compressed under pressure prior to introduction of the aqueous phase containing, for example, the recombinant protein. Polymeric particles then form of typical size range 300 nm–3 μm.

An ongoing concern with the introduction of NPs into biomedical applications has been their potential toxicity, not least because some materials which would otherwise be considered safe take on different characteristics in a nanoparticulate form and can sometimes become harmful (NIOSH, 2006). For example, in its naturally occurring mineral state titanium dioxide is biologically inert, however, when administered as a NP smaller than 20 nm in diameter it causes an inflammatory reaction in animals and humans (Ophus et al., 1979; Oberdorster et al., 1994). Similarly gold is generally regarded as a safe, inert material, and is used routinely for medical implants, however, gold NPs with a diameter of 1.4 nm behave very differently and have been shown to permeate cells and nuclear membranes and bind irreversibly in the major grooves of DNA causing instability (Tsoli et al., 2005). The same is not true of all gold NPs and those of a slightly larger diameter (15 nm) are considered non-toxic at up to 60-fold higher concentrations (Connor et al., 2005; Shukla et al., 2005; Pan et al., 2007; Villiers et al., 2010).

Other toxicity concerns associated with NP is the accumulation within cells, particularly with continuous exposure or long-term use. Indeed, fluorescent quantum dots have been observed in mice 2 years after injection (Fitzpatrick et al., 2009). As previously mentioned, the methods used to characterize cellular trafficking of NPs are often carried out using pharmacological inhibitors or mutant cell lines. The problem with these experiments is that seldom are these methods specific for one mechanism of trafficking so the data can often be difficult to interpret or sometimes contradictory to other literature. For example, whilst Rejman et al. show that treating B16 cells with chlorpromazine strongly inhibits the uptake of negatively charged 50 nm polystyrene NPS compared with 200 nm particles, dos Santos et al. report an opposite result (Rejman et al., 2004; Dos Santos et al., 2011). In many cases it is perhaps best to use a combination of various inhibitors and mutated cell lines with carefully selected controls. Another problem associated with pharmacological inhibitors is their cell line specific efficacy, meaning that care must be taken when interpreting results from such studies and perhaps highlights the need for using several different cell lines to draw conclusions applicable to an *in vivo* model.

There are also some more specific concerns over components used in NPs. Despite the number of veterinary vaccines which utilize ISCOMs, there are uncertainties over the toxicity of saponin-based adjuvants and this has to date prevented their licensure for use in humans. When administered intravenously, Quil A- derived ISCOMs and free Quil A are toxic to rats, and some mouse strains, at an LD_50_ of 0.67 mg/kg (Wünscher, 1994). Similar results have also been documented with subcutaneous and intraperitoneal administration of Quil A (Pyle et al., 1989; Stieneker et al., 1995) where it is believed to cause degeneration of the liver (Kersten et al., 1988). However, this has so far only been documented in rodents, with little toxicity reported in larger terrestrial animals including rhesus monkeys, chickens, dogs, or cattle (Vanselow et al., 1985; De Vries et al., 1994; Ma et al., 1995; Sundquist et al., 1996).

## Discussion

A wide variety of NP delivery systems have been described, each offering advantages over current methods of vaccine delivery. Rather than conventional vaccines which use whole microbes (live or killed), this new generation of vaccines use components of microbes to elicit an immune response and mimic the way in which these antigens would be delivered during a natural infection. Often these antigens are poor immunogens on their own and thus require an adjuvant to boost the immune response. Although previously demonstrated with alum-based adjuvants, this often fails to induce a cellular immune response and can be reactogenic toward the host. NPs provide an alternate method for antigen delivery which not only activates different elements of the immune system but also have good biocompatibility. One of the ways in which NPs are able to elicit different immune responses is through their size; moving into cells via non-classical pathways and then processed as such. Delivering antigens in different ways also has a profound effect on the resulting immune response, whether the antigen is decorated on the NP surface for presentation to antigen-presenting cells or encapsulated for slow release and prolonged exposure to the immune system. NPs are also versatile and can be modified with immunostimulatory compounds to enhance the intensity of the immune response or with molecules to increase their stability *in vivo* (polyethylene glycol).

Many of the NP delivery systems mentioned in this review are capable of eliciting both cellular and humoral immune responses. However, an efficient and protective vaccine is likely to induce a combination of both responses and should be tailored to the pathogen in question accordingly. Whilst these delivery vehicles may present as an exciting prospect for future vaccination strategies, it is also worth noting their potential drawbacks, particulary those associated with cytotoxicity. Since NPs have a relatively short history in medicine they do not have a longstanding safety profile in human use. It is therefore essential that further research is carried out in NP toxicity to fully address these questions if they are to be accepted as an alternative method for the delivery of novel vaccines and are licensed more widely for human use.

### Conflict of interest statement

The authors declare that the research was conducted in the absence of any commercial or financial relationships that could be construed as a potential conflict of interest.
